# [Corrigendum] Upregulation of centromere protein M promotes tumorigenesis: A potential predictive target for cancer in humans

**DOI:** 10.3892/mmr.2024.13186

**Published:** 2024-02-21

**Authors:** Ying Liu, Wenfeng Yu, Peng Ren, Ting Zhang

Mol Med Rep 22: 3922–3934, 2020; DOI: 10.3892/mmr.2020.11461

Subsequently to the publication of this paper, the authors’ have realized that Fig. 2A on p. 3927 was published featuring an error; specifically, there was an unintentional duplication of one of the representative images chosen for the figure (the same image was selected to represent the ‘BRCA/Normal’ and ‘Tumor/LUSC’ experiments). Additionally, the sample numbers in Fig. 2A were also incorrect. The correct sample numbers are as follows: 3 samples of breast tissue, 12 samples of breast cancer tissue, 3 samples of normal cervical tissue, 9 samples of CESC, 7 samples of LIHC, 3 samples of normal lung tissue and 6 samples of LUSC. These errors were due to negligence during the storage of HPA database images.

The revised version of Fig. 2, showing the correct data for the ‘Tumor/LUSC’ experiment in Fig. 2A (where the error occurred), is shown on the next page. Note that this error did not significantly affect either the results or the conclusions reported in this paper, and all the authors agree to the corrigendum. The authors do stress the importance of a larger sample size to ascertain statistically significant differences in CENPM protein expression, predominantly localized in the cell nucleus. Furthermore, the authors thank the Editor of *Molecular Medicine Reports* for allowing them the opportunity to publish this corrigendum, and apologize to the readership for any inconvenience caused.

## Figures and Tables

**Figure 2.**
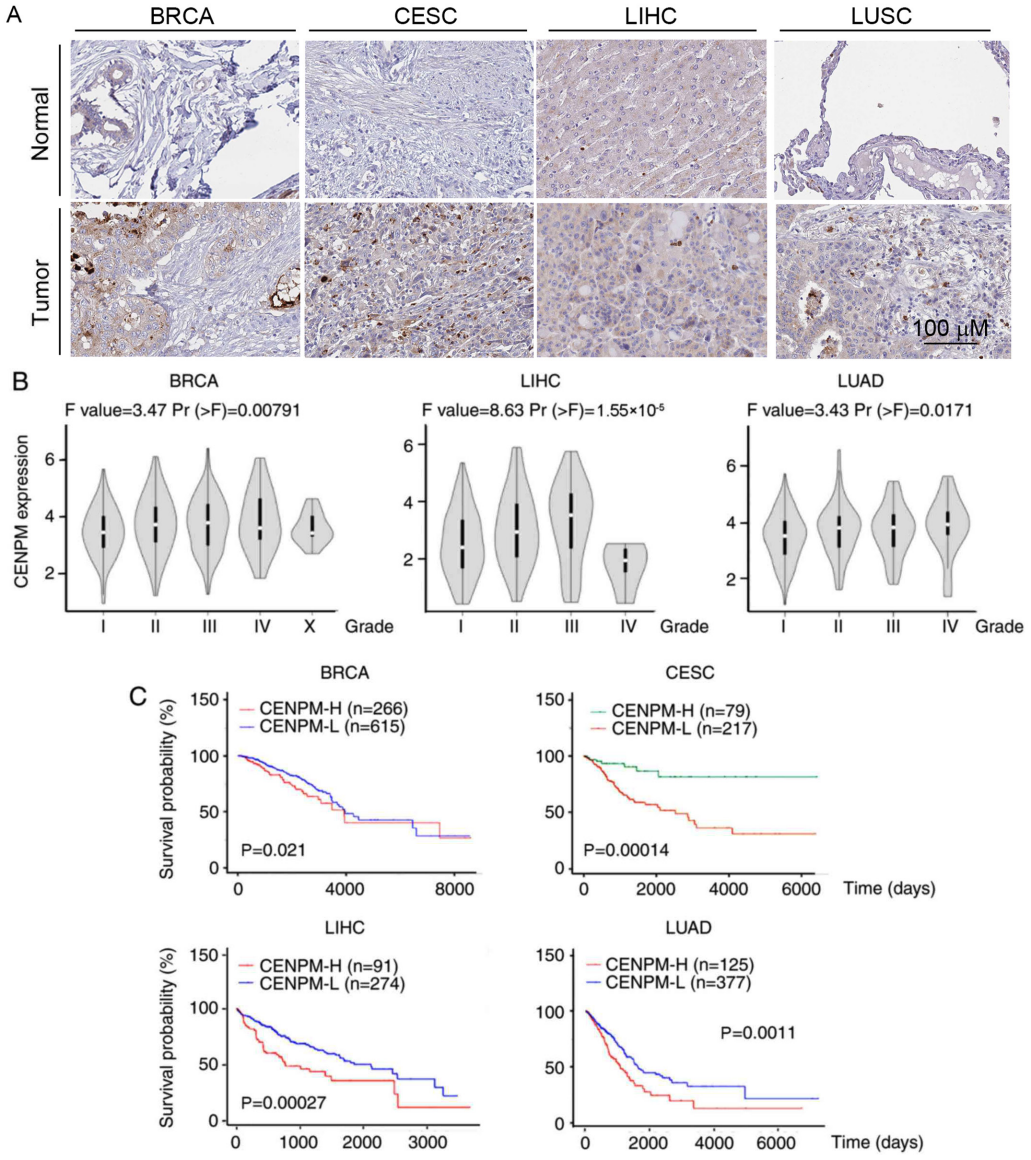
Expression levels of CENPM in various types of human cancer. (A) Immunohistochemical staining of CENPM protein levels in breast (n=2 for normal breast tissue and n=2 for BRCA tissue), cervix (n=2 for normal cervical tissue and n=2 for CESC tissue), liver (n=3 for normal liver tissue and n=1 for LIHC tissue) and lung (n=2 for normal lung tissue, and n=4 for LUSC tissue) samples. The data for the samples, which were collected and stained with a CENPM antibody, were obtained from the Human Protein Atlas. Scale bars, 200 µm. (B) Data on CENPM mRNA expression levels in BRCA, LIHC, and LUAD of different stages were downloaded from the Gene Expression Profiling Interactive Analysis database. (C) Kaplan-Meier plotter analysis of 881 patients with BRCA, 296 patients with CESC, 365 patients with LIHC and 502 patients with LUAD who had high or low CENPM mRNA expression. CENPM, centromere protein M; BRCA, invasive breast carcinoma; CESC, cervical squamous cell carcinoma and endocervical adenocarcinoma; LIHC, liver hepatocellular carcinoma; LUSC, lung squamous cell carcinoma; LUAD, lung adenocarcinoma.

